# Amelioration of renal injury by Qihuang Jianpi Zishen Granules in lupus mice is correlated with AMPK/ULK1-dependent modulation of macrophage polarisation

**DOI:** 10.1136/lupus-2025-001639

**Published:** 2025-10-29

**Authors:** Ai Qian, Kexin Hu, Yawen Zhu, Yuanyuan Cheng, Ming Li, Chuanbing Huang

**Affiliations:** 1 The First Affiliated Hospital of Anhui University of Traditional Chinese Medicine, Hefei, China

**Keywords:** Cytokines, Inflammation, Lupus Erythematosus, Systemic, Lupus Nephritis

## Abstract

**Objective:**

To investigate the effects of Qihuang Jianpi Zishen Granules (QJZG) on renal injury in SLE mice, focusing on macrophage M1/M2 polarisation mediated by the AMPK/ULK1 signalling pathway.

**Methods:**

Parameters of renal function and proteinuria were assessed. Pathological changes in the kidney were examined using H&E, periodic acid-Schiff and Masson’s trichrome staining. Serum inflammatory factor levels were quantified using ELISA. The expression levels of the glycolysis rate-limiting enzymes hexokinase 2 (HK2) and glucose transporter 1 (GLUT1) were determined, and the transcriptional levels of AMPK/ULK1 pathway components were measured using quantitative PCR. The abundance of proteins associated with AMPK/ULK1 signalling was assessed via immunoblotting. Flow cytometry was used to quantify CD86+ M1 type and CD206+ M2 type macrophage populations. Dual immunofluorescence staining was employed to visualise F4/80+CD86+ and F4/80+CD206+ coexpression patterns.

**Results:**

Compared with the Untreated group, mice in the PRED (prednisone acetate), QJZG and 2-Deoxy-D-glucose groups exhibited improved renal histopathology, reduced levels of creatinine, blood urea nitrogen, 24-hour RRO (24-hour urinary protein), ACR (Albumin-to-Creatinine Ratio), TPCR (Urine Total Protein-to-Creatinine Ratio), tumour necrosis factor alpha, interleukin (IL)-1β, IL-12, IL-23, IL-27, HK2, GLUT1, mTOR, CD86 and iNOS messenger RNA (mRNA), CD86 and iNOS proteins, M1 macrophages, M1/M2 macrophages and F4/80+CD86 expression (p<0.05). They also displayed increased expression of transforming growth factor-beta, IL-4, IL-10, C-C motif chemokine ligand 18, AMPK, ULK1, Atg13, CD206 and Arg-1 mRNA, AMPK, ULK1, CD206 and Arg-1 proteins, M2 macrophages and F4/80+CD206 (p<0.05).

**Conclusion:**

QJZG effectively improved renal injury in SLE by reducing inflammation and modulating the AMPK/ULK1 signalling pathway to suppress M1 macrophage polarisation.

**WHAT IS ALREADY KNOWN ON THIS TOPIC**

- Effective Western medicine treatments for lupus nephritis (LN) remain limited. While Qihuang Jianpi Zishen Granule (QJZG) demonstrates protective effects against renal inflammation and fibrosis, its therapeutic potential for LN-associated renal damage is unclear.

**WHAT THIS STUDY ADDS**

- We found that QJZG alleviates renal injury, improves renal function and reduces proteinuria in a murine lupus model. Mechanistically, it suppresses renal inflammation by modulating AMPK/ULK1-mediated macrophage polarisation, particularly by inhibiting M1 macrophage glycolysis.

**HOW THIS STUDY MIGHT AFFECT RESEARCH, PRACTICE OR POLICY**

- This study identifies QJZG as a promising candidate for LN treatment, warranting further clinical investigation.

## Introduction

 Lupus nephritis (LN), a common complication in SLE characterised by proteinuria, microscopic haematuria, urinary casts and progressive renal dysfunction, occurs in over 50% of patients with SLE. Renal biopsies in nearly all patients with SLE reveal pathological changes of varying severity.^1^ The pathogenesis and molecular mechanisms underlying SLE are highly complex, and the specific pathways through which SLE leads to renal damage are still not fully elucidated. Therefore, identifying new therapeutic targets and treatment strategies remains critical.

Previous studies have demonstrated that Qihuang Jianpi Zishen Granules (QJZG) can reduce the levels of various inflammatory cytokines, improve immune regulation and exert therapeutic effects on SLE-induced renal damage.^2^,^4^ Previous animal studies have demonstrated that QJZG mitigates the proliferation of SLE mesangial cells by modulating the GAS5/miR-21/SIRT1 axis and suppressing the ERK/CREB pathway.^5^ Separately, Li *et al*^6^ investigated the role of the Ca^2+^/CaMKK2/AMPK/mTOR signalling pathway in autophagy regulation, particularly focusing on how QJZG ameliorates thrombocytopenia in SLE mice. In contrast, our study advances the understanding of SLE-associated kidney injury by uncovering a novel mechanism involving macrophage metabolic reprogramming. We propose that this work not only offers a fresh perspective but also establishes a new experimental framework, thereby extending the field beyond our earlier contributions.

Macrophages, as essential phagocytes in immune responses and central regulators of inflammation, undergo significant metabolic reprogramming in response to microenvironmental changes and as such, they play a crucial role in SLE-induced renal injury.^7 8^ Research suggests that the AMPK/ULK1 signalling pathway can suppress the mTOR/HIF-1α axis, downregulate key glycolytic enzymes, such as hexokinase 2 (HK2) and glucose transporter 1 (GLUT1), and eventually inhibit M1 macrophage polarisation.^9 10^ However, it remains unclear whether QJZG can mitigate SLE-induced renal injury by modulating macrophage M1/M2 polarisation through this signalling pathway. Hence, using animal experiments, this study aimed to elucidate the specific mechanisms by which QJZG alleviates SLE-induced renal injury, identify potential therapeutic targets and provide scientific evidence to support its clinical application and broader implementation.

## Materials and methods

### Materials

#### Animals

24 female MRL/lpr lupus mice (8–9 weeks old) and six C57BL/6 mice (used as the control group) were obtained from the Anhui Laboratory Animal Center (Licence No. SCXK (Hu) 2022–0004) and housed in a specific pathogen-free (SPF)-level animal room. The mice were allowed to acclimatise for 1 week, during which they were provided with sterilised specialised feed and had ad libitum access to water, before initiating the experiments.

#### Drugs

The following drugs were used in this study: QJZG (10 g per bag, Approval No. 20220041000, an in-house preparation of the First Affiliated Hospital of Anhui University of Chinese Medicine). A total of 114 chemical compounds were identified from QJZG using UPLC-Q-TOF-MSE coupled with UNIFI software and manually verified with MassLynx V.4.1. The compounds originated from the following herbal constituents: 18 from Astragalus membranaceus, 27 from Rehmannia glutinosa, 19 from Cuscuta chinensis, 9 from yam, 5 from Atractylodes macrocephala, 14 from Rosa rugosa, 17 from raspberry and 18 from Poria cocos, with 11 compounds being common to multiple herbs. The major chemical classes identified in QJZG included flavonoids, terpenoids, organic acids, phenylethanoid glycosides, saponins and lignans. Furthermore, using the established compound library of 114 chemical constituents as potential in vivo prototypes, a total of 27 prototype compounds and 51 metabolites were characterised through the same analytical platform complemented by manual validation. Flavonoids and terpenoids were identified as the predominant prototype components. The major metabolic pathways involved were hydrolysis, decarboxylation, methylation and related biotransformations.^11^

2-Deoxy-D-glucose (2-DG) (Catalogue No. HY-13966, provided by Shanghai Haoyuan Biomedical Technology). 2-DG, a glucose analogue, functions as a competitive inhibitor of HK2. It disrupts glucose metabolism and suppresses glycolysis without impairing oxidative phosphorylation. As a well-established reference inhibitor in this field, 2-DG ensures experimental reproducibility and facilitates cross-study comparisons. Therefore, this study employs 2-DG to simulate the activation of the AMPK/ULK1 pathway.^12 13^

Prednisone acetate tablets (5 mg per tablet, National Drug Approval No. H12020689, manufactured by Tianjin Tianyao Pharmaceutical).

#### Reagents

Creatinine (CREA), blood urea nitrogen (BUN), 24-hour urinary protein (24-hour PRO), Albumin-to-Creatinine Ratio (ACR), Urine Total Protein-to-Creatinine Ratio (TPCR), tumour necrosis factor alpha (TNF-α), interleukin (IL)-1β, IL-12, IL-23, IL-27, HK2 and GLUT1 markers of renal function and inflammation (Wuhan GeneMay Biotechnology); Primers synthesised by Sangon Biotech; AMPK-a (CST), ULK1 (Bioworld), CD86 (bioss), iNOS (bioss), CD206 (Santa Cruz), Arg-1 (Proteintech) and GAPDH (Zsbio) antibodies; HE, periodic acid-Schiff (PAS) and Masson (ebiogo) staining kits; F4/80 (Wuhan Sanying), CD86 (Bioss) and CD206 antibody (Wuhan Sanying) for immunohistochemistry.

#### Instruments

CytoFLEX Flow Cytometer (Beckman), Pannoramic MIDI Digital Slide Scanner (3DHISTECH, Hungary), K90 Standard PCR machine (Hangzhou Jingge Scientific Instrument), PIKOREAL 96 Real-Time PCR System (Thermo Scientific), EPS300 Electrophoresis System (Tanon), JW-3021HR High-Speed Refrigerated Centrifuge (Anhui Jiawen Instrument Equipment), ZT-12M Automatic Tissue Dehydrator and YB-7LF Tissue Embedding Machine (Xiaogan Yaguang Medical Electronics Technology).

### Methods

#### Animal grouping and drug administration protocol

The 24 SPF-grade female MRL/lpr lupus mice were randomly assigned to one of the following four groups (n=6 per group): an untreated (Untreated) group, a prednisone acetate (PRED) group, a QJZG group and a glycolysis inhibitor (2-DG) group. In addition, the six C57BL/6 mice were used as the normal control (Control) group. For each group, besides the routine feeding, the animals were treated with the required drugs, with the doses calculated based on equivalent body surface area across species. The treatment plan was as follows: The Control and Untreated groups received normal saline via gavage at a dose of 10 mL/kg/day, while for the PRED group, the mice were administered prednisone acetate tablets (dissolved in normal saline to a final concentration of 0.273 mg/mL) via gavage at a dose of 2.73 mg/kg/day (equivalent to an adult dose of 0.3 mg/kg/day). Additionally, for the QJZG group, QJZG was dissolved in normal saline to a concentration of 0.39 g/mL solution before being administered by gavage at a dose of 3.9 g/kg/day (equivalent to an adult dose of 0.43 g/kg/day). Finally, in the case of the 2-DG group, the animals were given normal saline by gavage (10 mL/kg/day^−1^), along with intraperitoneal injections of 2-DG at 2 mg/kg^−1^ every other day. All treatments were continuously administered for 8 weeks.

#### Assessment of renal function indicators

Renal function was assessed by measuring the levels of CREA, BUN, 24-hour PRO, ACR and TPCR in each group of mice. For this purpose, serum and urine samples were first collected before being analysed as follows: serum CREA concentrations were determined using an enzymatic sarcosine oxidase assay; blood BUN levels were measured using the urease method; urinary 24-hour PRO levels were assessed with the Coomassie Brilliant Blue method; urinary ACR and TPCR levels were measured using the microplate assay method.

#### Renal pathological examination

Kidney tissues were fixed, paraffin-embedded and sectioned, after which the sections were dewaxed, dehydrated through a graded ethanol series and cleared with xylene. This was followed by H&E, PAS and Masson staining which were performed according to standard protocols. Slides with the stained sections were eventually mounted with neutral resin and examined under a microscope.

#### Quantification of circulating inflammatory mediators was quantified through ELISA technique

The concentrations of circulating inflammatory mediators were quantified using ELISA technique. Following the treatment period, mice were fasted for 8 hours prior to blood collection. Venous blood samples were then collected and centrifuged at 2500 rpm for 5 min to obtain serum, which was subsequently stored at −80°C to minimise degradation from freeze-thaw cycles. Serum levels of TNF-α, IL-1β, IL-12, IL-23, IL-27, transforming growth factor-beta (TGF-β), IL-4, IL-10 and C-C motif chemokine ligand 18 (CCL-18) were eventually measured in accordance with the ELISA kit protocols.

#### Measurement of key glycolytic enzymes (HK2, GLUT1)

Blood samples were allowed to coagulate at room temperature for 10–20 min, after which a 20 min centrifugation was performed at 2000–3000 rpm. The resulting supernatant was then carefully collected, ensuring the absence of any precipitates during storage. HK2 levels were subsequently measured by spectrophotometry, while GLUT1 expression was quantified using ELISA based on the dual-antibody sandwich method.

#### RT-qPCR-based analysis of mRNA expression in kidney tissues

Total RNA was extracted from 50 to 100 mg of kidney tissues using the TRIzol reagent. Reverse transcription was subsequently performed, during which genomic DNA was first removed prior to centrifugation. The samples were then heated at 42℃ for 2 min in a PCR instrument, after which they were briefly cooled on ice for 1 min. This was followed by enzymatic reactions, carried out under temperature-controlled conditions (37℃/15 min→85℃/5 s), with the resulting complementary DNA eventually stored at −80℃. In this experiment, PCR amplification was performed in 10 µL reaction volumes under the following conditions: initial denaturation at 95℃ for 1 min, followed by 40 cycles, each involving denaturation for 20 s at 95℃, followed by annealing at 60℃ for 1 min. Finally, the relative messenger RNA (mRNA) expression of *AMPK*, *ULK1*, *mTOR*, *Atg13*, *CD86*, *iNOS*, *CD206* and *Arg-1* in kidney tissues was analysed using the 2^-△△Ct^ method. The results included amplification curves, melting curves as well as relative expression values. The primer sequences used for this set of experiments are shown in table 1.

**Table 1:** Primers for RT-qPCR test

Gene	Amplicon size (bp)	Forward primer (5'→3')	Reverse primer (5'→3')
*β-actin*	120	AGTGTGACGTTGACATCCGT	TGCTAGGAGCCAGAGCAGTA
*AMPK*	92	AGTGAAGACTACCAGGTGAT	TTGCAGATGTAGTCGAACAA
*ULK1*	140	CACCCTTTTCCTACCAGTG	CAGTGGTTCTTGGAGAGTG
*CD86*	102	GAAAGAGGAGCAAGCAGACG	TGGGTGCTTCCGTAAGTTCT
*iNOS*	94	GGAGCGAGTTGTGGATTGTC	CAGCCTCTTGTCTTTGACCC
*CD206*	110	AGTGGCTTTGGTTGAACGAC	CCAAAGGCCCGAAGATGAAG
*Arg-1*	135	GCAGTTGGAAGCATCTCTGG	GAGAAAGGACACAGGTTGCC
*mTOR*	156	ATTGACTTTGGGGACTGCTT	GAGCACTTCCATCACGGT
*Atg13*	82	AGACTCTCAGGAAGTGTGTA	AAAATGTGCCTTTGTGACAG

RT-qPCR, reverse-transcription quantitative PCR.

#### Western blot analysis of protein expression in kidney tissues

The protein expression levels of AMPK, ULK1, CD86, iNOS, CD206 and Arg-1 in kidney tissues were determined using western blotting. For this purpose, renal cortical tissues were mechanically homogenised for extracting proteins, which were then separated by Sodium Dodecyl Sulfate - Polyacrylamide Gel Electrophoresis (80 V, 1 hour) before being transferred to membranes. These membranes were subsequently blocked for 120 min prior to a 16-hour incubation with the primary antibodies at 4℃. After washing, the membranes were incubated with Horseradish Peroxidase (HRP)-conjugated secondary antibodies at room temperature for 72 min. Protein bands were finally visualised using Enhanced ChemiLuminescence (ECL) substrate.

(80 V, 1 hour) before being transferred to membranes. These membranes were subsequently blocked for 120 min prior to a 16-hour incubation with the primary antibodies at 4℃. After washing, the membranes were incubated with HRP-conjugated secondary antibodies at room temperature for 72 min. Protein bands were finally visualised using ECL substrate.

#### Flow cytometric analysis of CD86+M1 and CD206+M2 macrophage populations in kidney tissues

Kidney tissues were mechanically homogenised to prepare single-cell suspensions, which were then washed twice with phosphate-buffered saline (PBS) prior to a 10 min centrifugation at 3500 rpm. The resulting pellet was resuspended in PBS, and 200 µL of the suspension was incubated with 5 µL of CD86 antibody at room temperature in the dark for 15 min. The cells were then fixed and permeabilised before adding 5 µL each of CD68 and CD206 antibodies. Following a 20 min incubation in the dark at room temperature, the cells were washed with PBS, resuspended and eventually analysed by flow cytometry. In this case, the data were processed using FlowJo_V10 software.

#### Immunofluorescence analysis of M1 and M2 macrophage spatial distribution in kidney tissues

The spatial localisation of M1-polarised and M2-polarised macrophages in kidney tissues was assessed by dual immunofluorescence labelling using distinct fluorophores. In this case, tissue sections were dewaxed using xylene and then rehydrated through graded ethanol before being washed for removing ethanol. Antigen retrieval was then performed using citrate buffer under high pressure, after which the sections were incubated with the primary antibodies for 16 hours at 4°C. This was followed by incubation with fluorophore-conjugated secondary antibodies at 37°C for 30 min. The slides were finally coverslipped and scanned using a Pannoramic MIDI digital slide scanner to capture fluorescence images.

#### Statistical analysis

The results, expressed as mean±SD, were statistically analysed using SPSS V.26.0 software package. Comparisons between two groups were performed using Student’s t-test, while multiple-group comparisons were conducted using one-way analysis of variance. For all analyses, statistical significance was considered at p<0.05. In addition, graphical illustrations were prepared with GraphPad Prism and ImageJ software.

## Results

### Effects of QJZG on CREA, BUN, 24-hour PRO, ACR and TPCR levels in MRL/lpr mice

**Figure 1:**
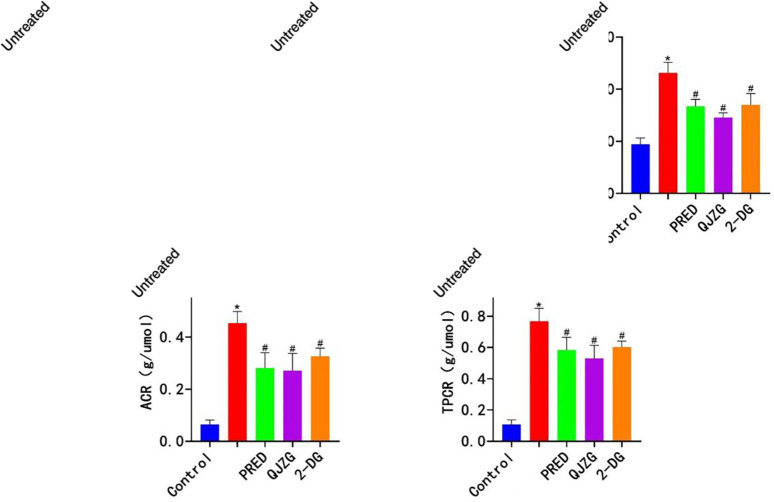
(A) Positive expression of CREA. (B) Positive expression of BUN. (C) Positive expression of 24-hour PRO. (D) Positive expression of ACR. (E) Positive expression of TPCR. ^*^Indicates significant differences compared with the Control group (p<0.05); ^#^denotes significant differences relative to the Untreated group (p<0.05). BUN, blood urea nitrogen; Control, control group; CREA, creatinine; 2-DG, 2-Deoxy-D-glucose group; PRED, prednisone acetate group; QJZG, Qihuang Jianpi Zishen Granules group; Untreated, untreated group.

Compared with the Control group, the Untreated group exhibited significantly elevated levels of serum CREA, BUN, urinary 24-hour PRO, ACR and TPCR (p<0.05). In contrast, the PRED, QJZG and 2-DG groups showed significant reductions in CREA, BUN, 24-hour PRO, ACR and TPCR levels compared with the Untreated group (p<0.05). These results are summarised in figure 1.

### Effects of QJZG on renal pathology in MRL/lpr mice

1. HE staining

In the Control group, the kidney tissues exhibited well-organised and distinct structural integrity, with uniform staining, neatly arranged renal tubules and no evidence of inflammatory cell infiltration. On the other hand, the Untreated group displayed evident pathological alterations, including irregular staining, abnormal glomerular structures, mesangial matrix expansion and basement membrane thickening. In addition, renal tubular epithelial cells showed multiple vacuolar degeneration and dilation, leading to a disrupted tubular arrangement, with interstitial oedema and inflammatory cell infiltration also observed. However, the PRED, QJZG and 2-DG treatments effectively prevented the development and progression of nephritis, as demonstrated by significant amelioration in renal histopathology. Morphological analysis revealed partial preservation of glomerular architecture and renal tubule organisation, a moderated increase in glomerular volume compared with the Untreated group, along with suppressed mesangial expansion, thinning of the basement membrane, reduced interstitial oedema and a marked decrease in inflammatory cell infiltration.

1. PAS staining

In the Control group, the kidney tissues displayed a well-organised architecture, with clearly-defined structures and no notable pathological abnormalities. However, in the Untreated group, pathological changes, such as disorganised tissue architecture, glomerular mesangial thickening and expansion, hyperplasia, inflammatory cell infiltration, cell necrosis and cellular crescents, were observed. Compared with the Untreated group, kidney tissue damage in the PRED, QJZG and 2-DG groups was significantly alleviated.

1. Masson staining

**Figure 2:**
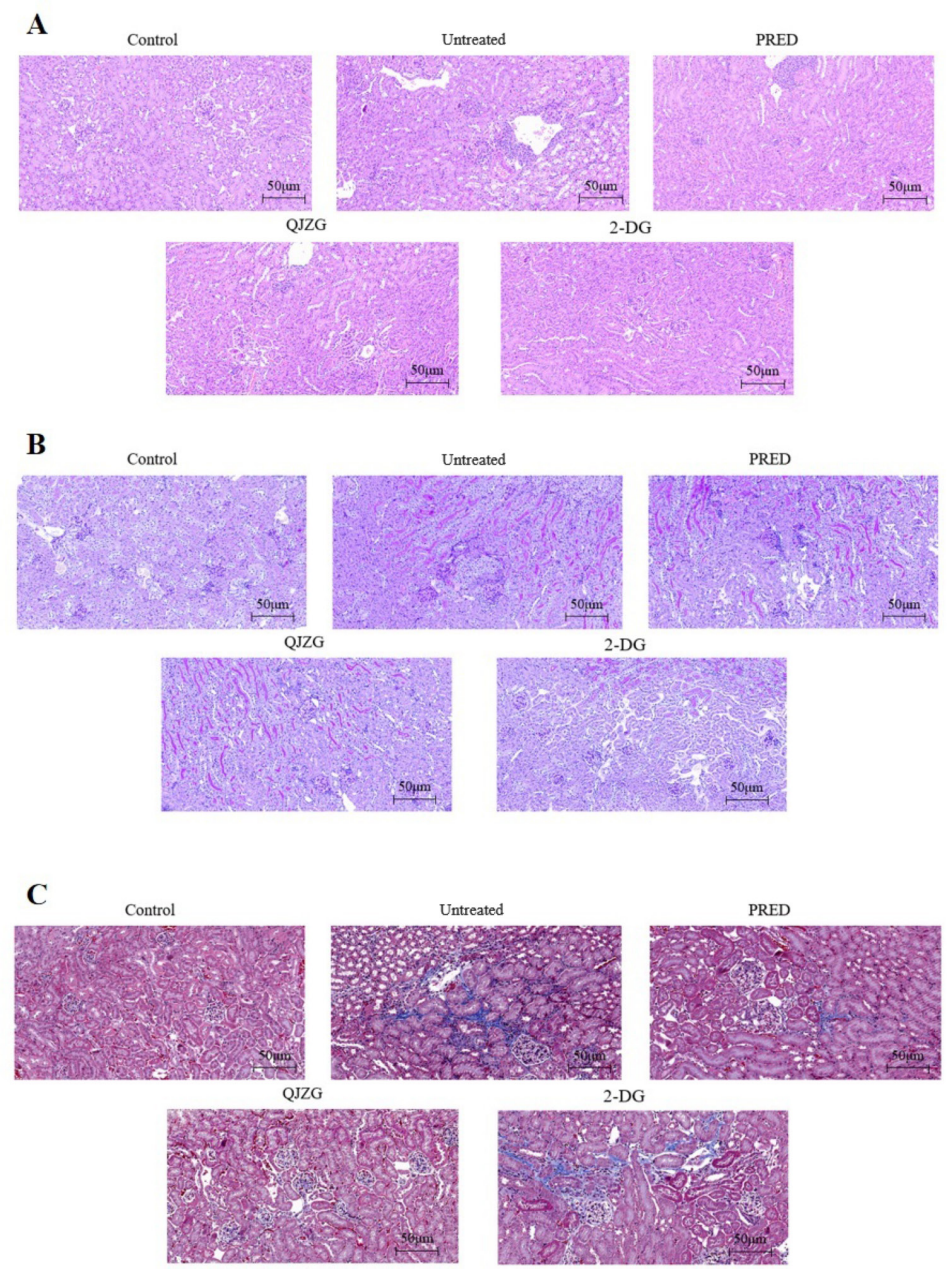
(A) Histopathological changes in kidney tissues from the different groups, shown by H&E staining (×200). (B) PAS-staining showing histopathological changes in the kidney tissues of the different groups (×200). (C) Pathological changes in mouse kidney tissues, shown by Masson staining (×200). Control, control group; 2-DG, 2-Deoxy-D-glucose group; PAS, periodic acid-Schiff; PRED, prednisone acetate group; QJZG, Qihuang Jianpi Zishen Granules group; Untreated, untreated group.

In the Control group, the kidney tissues exhibited no significant signs of connective tissue hyperplasia, fibrosis or hardening. In contrast, the Untreated group displayed extensive and clustered blue-stained collagen fibre regions which indicated pronounced glomerular fibrosis. However, compared with the Untreated group, the PRED, QJZG and 2-DG groups showed a marked reduction in glomerular fibrosis (figure 2).

### Effects of QJZG on the levels of TNF-α, IL-1β, IL-12, IL-23 and IL-27 in MRL/lpr mice

**Figure 3:**
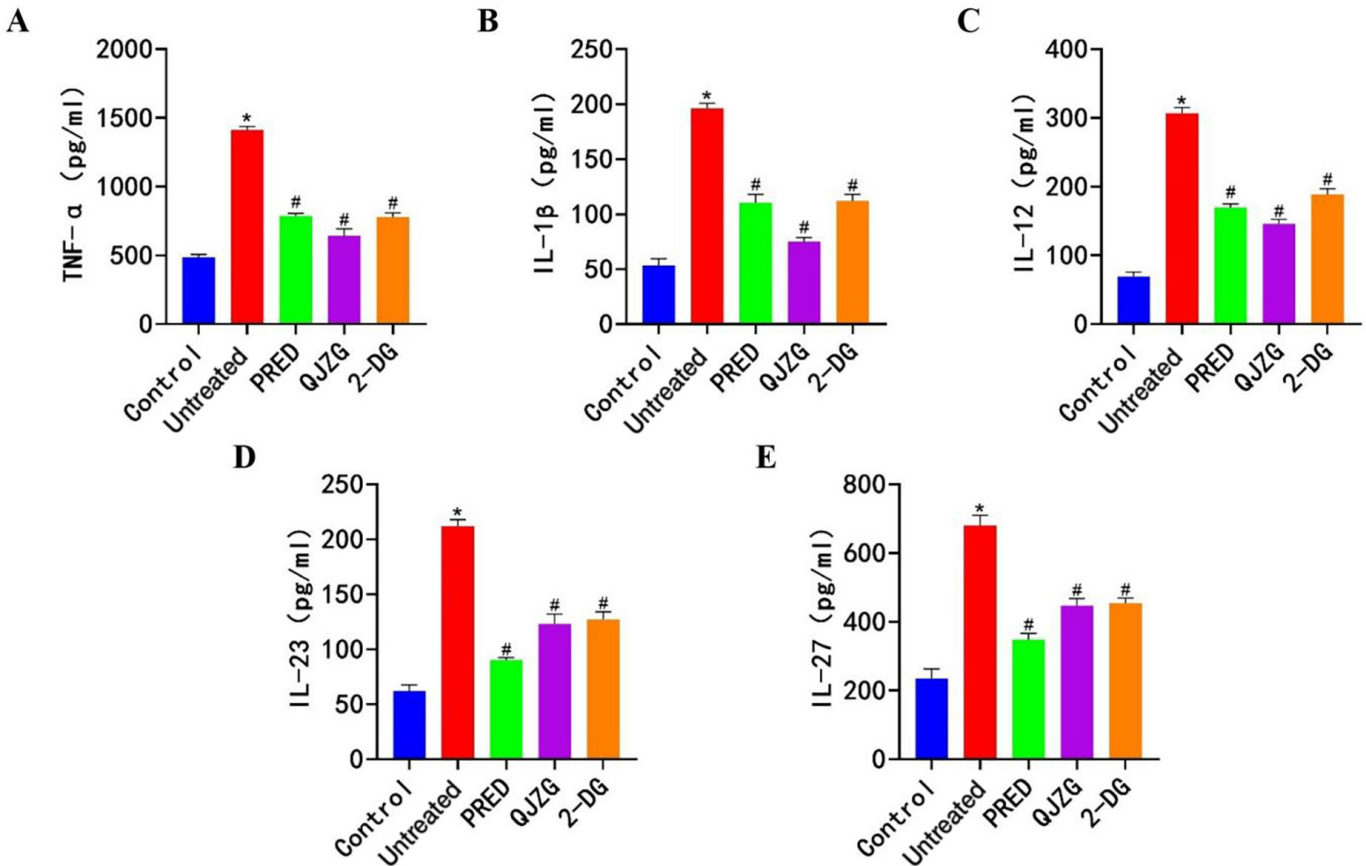
(A) Positive expression of TNF-α. (B) Positive expression of IL-1β. (C) Positive expression of IL-12. (D) Positive expression of IL-23. (E) Positive expression of IL-27. ^*^Indicates significant differences compared with the Control group (p<0.05); ^#^denotes significant differences relative to the Untreated group (p<0.05). Control, control group; 2-DG, 2-Deoxy-D-glucose group; IL, interleukin; PRED, prednisone acetate group; QJZG, Qihuang Jianpi Zishen Granules group; TNF-α, tumour necrosis factor alpha; Untreated, untreated group.

Compared with the Control group, the Untreated group showed significantly elevated serum levels of proinflammatory cytokines TNF-α, IL-1β, IL-12, IL-23 and IL-27 (p<0.05). However, the PRED, QJZG and 2-DG groups exhibited a significant reduction in the levels of these cytokines compared with the Untreated group (p<0.05), as presented in figure 3.

### Effects of QJZG on the levels of TGF-β, IL-4, IL-10 and CCL-18 in MRL/lpr mice

**Figure 4:**
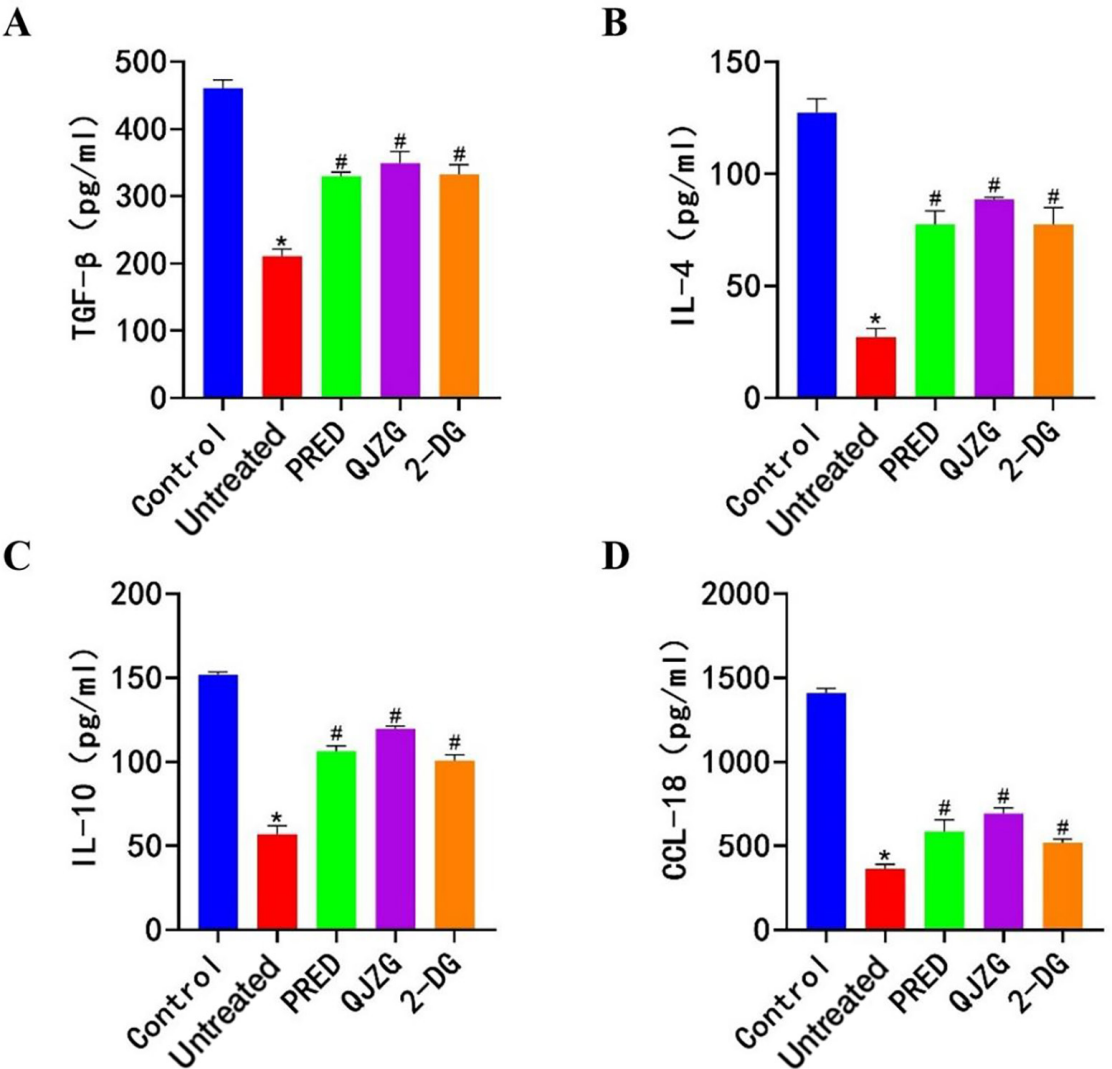
(A) Positive expression of TGF-β. (B) Positive expression of IL-4. (C) Positive expression of IL-10. (D) Positive expression of CCL-18. ^*^Indicates significant differences compared with the Control group (p<0.05); ^#^denotes significant differences relative to the Untreated group (p<0.05). CCL-18, C-C motif chemokine ligand 18; Control, control group; 2-DG, 2-Deoxy-D-glucose group; IL, interleukin; PRED, prednisone acetate group; QJZG, Qihuang Jianpi Zishen Granules group; TGF-β, transforming growth factor-beta; Untreated, untreated group.

Compared with the Control group, the Untreated group exhibited significantly lower serum levels of anti-inflammatory cytokines TGF-β, IL-4, IL-10 and CCL-18 (p<0.05). However, the PRED, QJZG and 2-DG groups showed a marked increase in the levels of these cytokines compared with the Untreated group (p<0.05), as shown in figure 4.

### Effects of QJZG on the levels of HK2 and GLUT1 in MRL/lpr mice

**Figure 5:**
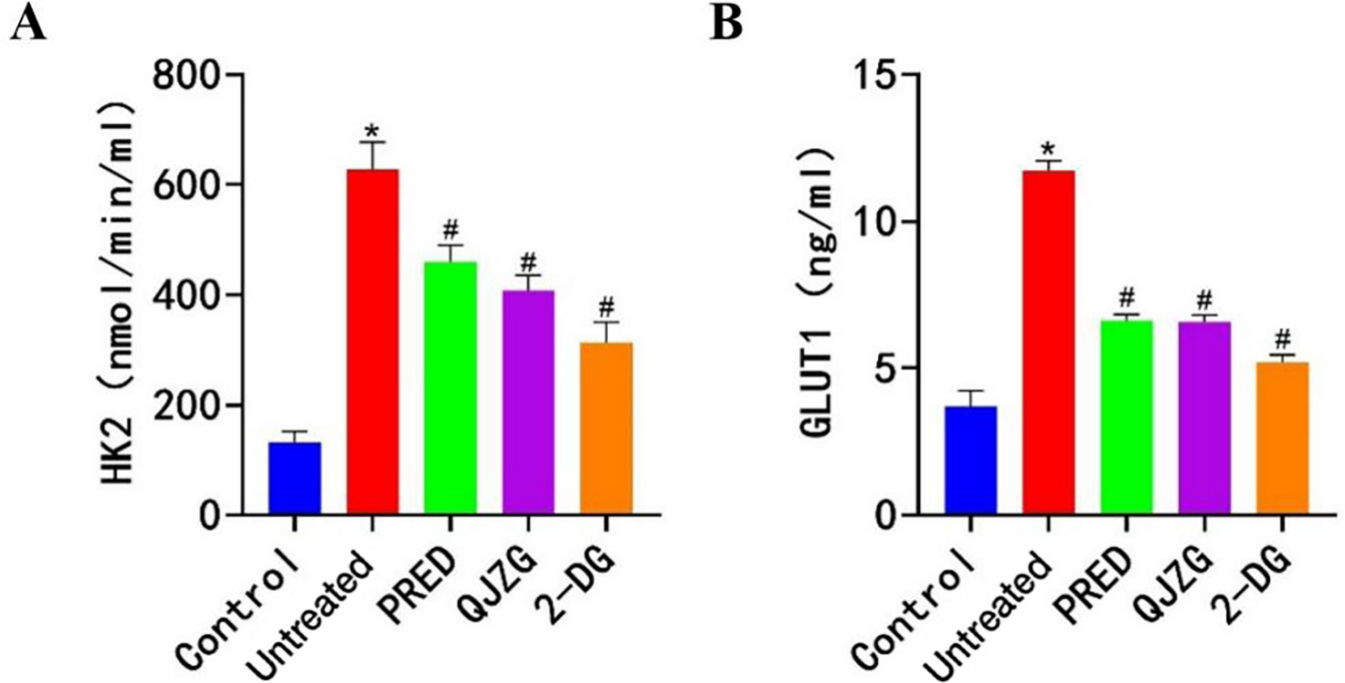
(A) Positive expression of HK2. (B) Positive expression of GLUT. ^*^Indicates significant differences compared with the Control group (p<0.05); ^#^denotes significant differences relative to the Untreated group (p<0.05). Control, control group; 2-DG, 2-Deoxy-D-glucose group; GLUT1, glucose transporter 1; HK2, hexokinase 2; PRED, prednisone acetate group; QJZG, Qihuang Jianpi Zishen Granules group; Untreated, untreated group.

Compared with the Control group, the Untreated group had significantly higher serum levels of HK2 and GLUT1 (p<0.05). However, compared with the Untreated group, the PRED, QJZG and 2-DG groups exhibited a significant reduction in HK2 and GLUT1 levels (p<0.05), as presented in figure 5.

### Effects of QJZG on the mRNA expression levels of *AMPK*, *ULK1*, *mTOR*, Atg13, *CD86*, *iNOS*, *CD206* and *Arg-1* in the kidneys of MRL/lpr mice

**Figure 6:**
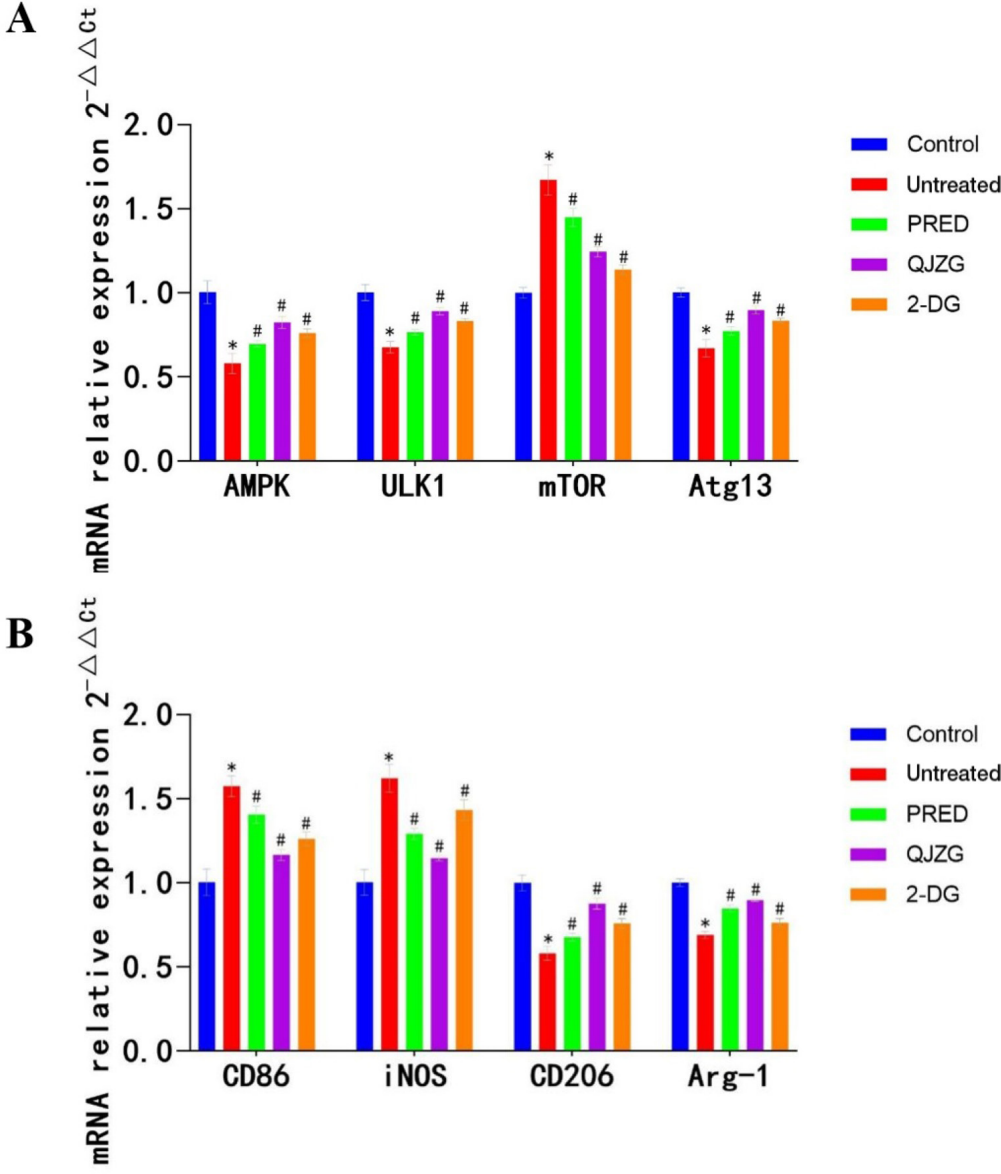
(A) Comparison of AMPK, ULK1, mTOR and Atg13 mRNA expression levels among the different groups. (B) Comparison of CD86, iNOS, CD206 and Arg-1 mRNA expression levels among the different groups. ^*^p<0.05 versus Control group; ^#^p<0.05 versus Untreated group. Control, control group; 2-DG, 2-Deoxy-D-glucose group; mRNA, messenger RNA; PRED, prednisone acetate group; QJZG, Qihuang Jianpi Zishen Granules group; Untreated, untreated group.

Compared with the Control group, mice in the Untreated group had significantly lower relative mRNA expression levels of *AMPK*, *ULK1*, *Atg13*, *CD206* and *Arg-1* in the kidney tissues (p<0.05). Conversely, the relative mRNA expression levels of *mTOR*, *CD86* and *iNOS* were notably increased (p<0.05). However, compared with the Untreated group, the PRED, QJZG and 2-DG groups exhibited a significant increase in the relative mRNA expression levels of *AMPK*, *ULK1*, *Atg13*, *CD206* and *Arg-1* in the kidney tissues (p<0.05), while the relative mRNA expression levels of *mTOR*, *CD86* and *iNOS* were notably reduced (p<0.05), as illustrated in figure 6.

### Effects of QJZG on the protein levels associated with the AMPK/ULK1 pathway in the kidneys of MRL/lpr mice

**Figure 7:**
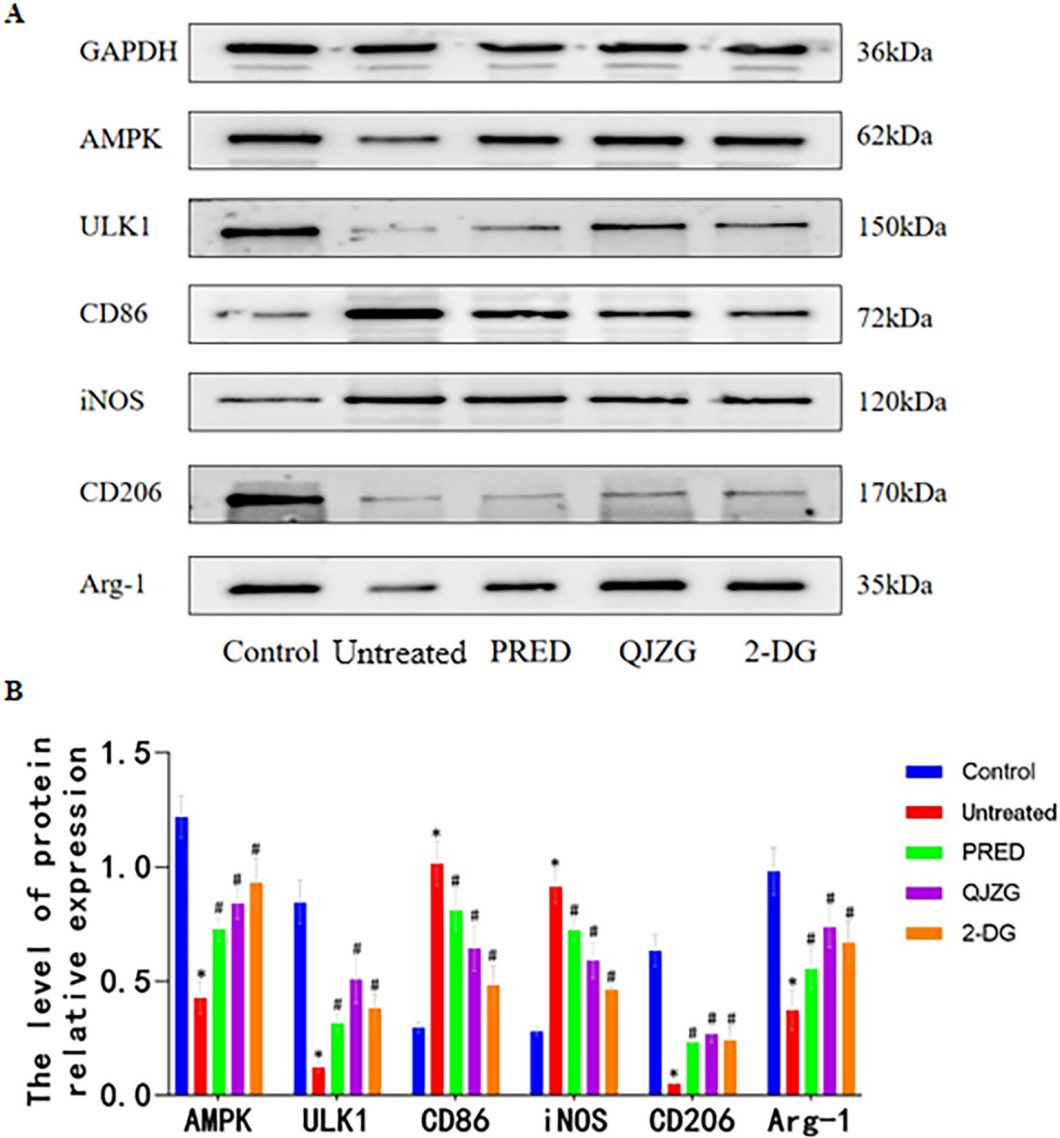
(A) Western blots showing the protein levels of AMPK, ULK1, CD206, Arg-1, CD86 and iNOS in the kidney tissues of lupus mice. (B) Comparison of AMPK/ULK1 pathway-related proteins in each group. ^*^p<0.05 versus Control group; ^#^p<0.05 versus Untreated group. Control, control group; 2-DG, 2-Deoxy-D-glucose group; PRED, prednisone acetate group; QJZG, Qihuang Jianpi Zishen Granules group; Untreated, untreated group.

Compared with the Control group, the Untreated group exhibited significantly lower relative expression levels of AMPK, ULK1, CD206 and Arg-1 proteins in the kidney tissues (p<0.05). In contrast, the relative expression levels of CD86 and iNOS proteins were markedly elevated (p<0.05). Furthermore, compared with the Untreated group, the PRED, QJZG and 2-DG groups showed a significant increase in the relative expression levels of AMPK, ULK1, CD206 and Arg-1 proteins in the kidney tissues (p<0.05). At the same time, the relative expression levels of CD86 and iNOS proteins were notably reduced (p<0.05), as illustrated in figure 7.

### Modulatory effects of QJZG on renal macrophage polarisation in MRL/lpr mice

**Figure 8:**
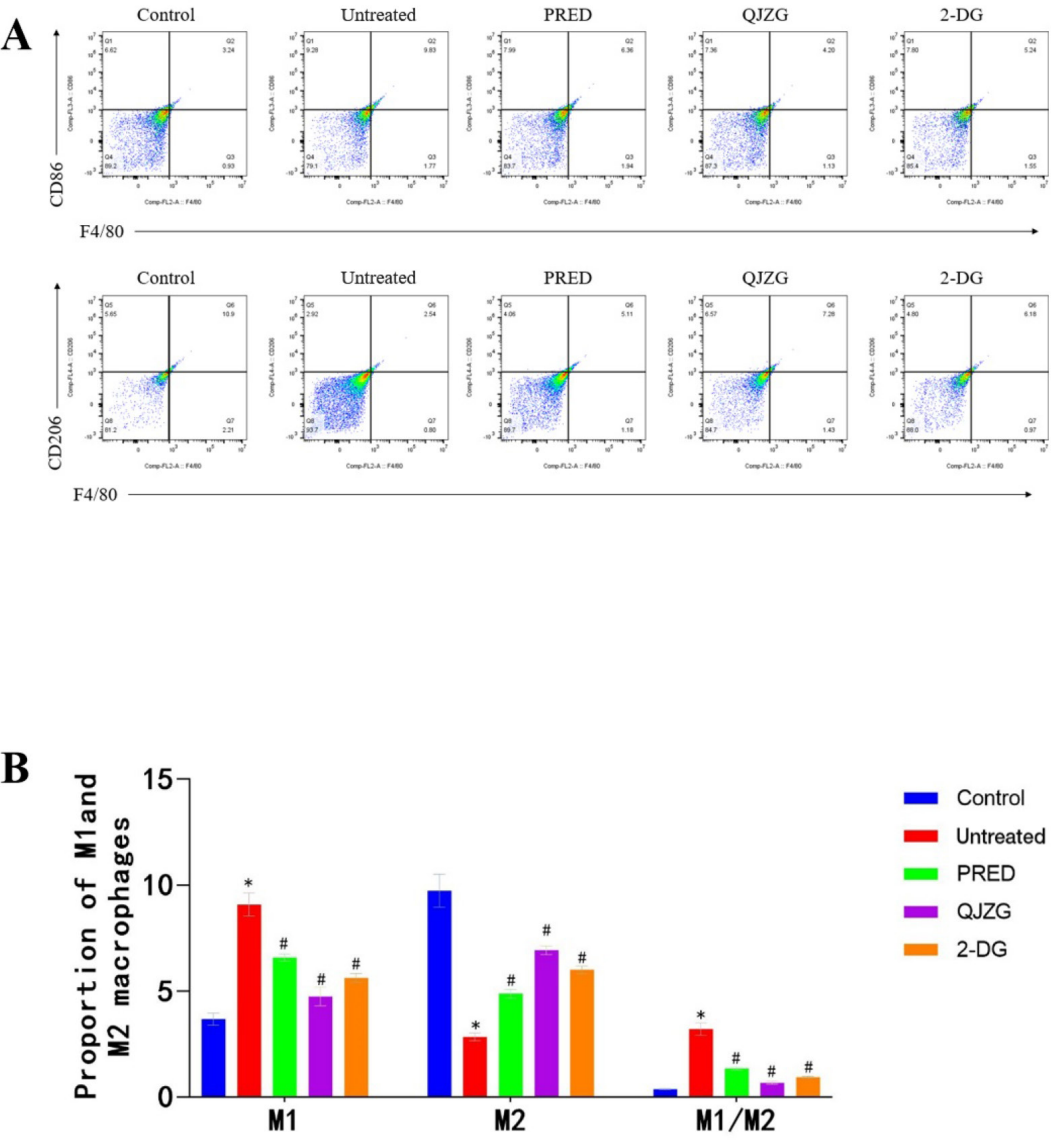
(A) Proportions of M1 and M2 macrophages in the kidney tissues of lupus mice, as determined by flow cytometry. (B) Quantitative comparison of M1 and M2 macrophage populations in kidney tissues among the different groups. ^*^Indicates significant differences compared with the Control group (p<0.05); ^#^denotes significant differences relative to the Untreated group (p<0.05). Control, control group; 2-DG, 2-Deoxy-D-glucose group; PRED, prednisone acetate group; QJZG, Qihuang Jianpi Zishen Granules group; Untreated, untreated group.

The modulatory effects of QJZG on renal macrophage polarisation were assessed through a quantitative analysis of CD86+M1 to CD206+M2 macrophage populations in MRL/lpr mice. Quantitative assessment revealed significant differences in macrophage polarisation across the different experimental groups. Overall, the Untreated group exhibited a markedly higher proportion of M1 macrophages (41.3% vs 12.5%, p<0.05), a lower proportion of M2 macrophages (18.7% vs 35.2%, p<0.05) and an increased M1/M2 ratio (2.21 vs 0.36, p<0.05) compared with the Control. However, treatment with PRED, QJZG or 2-DG significantly reversed these changes, leading to a reduction in M1 macrophage percentages (p<0.05), an increase in M2 macrophage populations (p<0.05) and a decrease in the M1/M2 ratio (p<0.05) compared with the Untreated group (figure 8).

### Effects of QJZG on F4/80+CD86 and F4/80+CD206 expression in MRL/lpr mice

**Figure 9:**
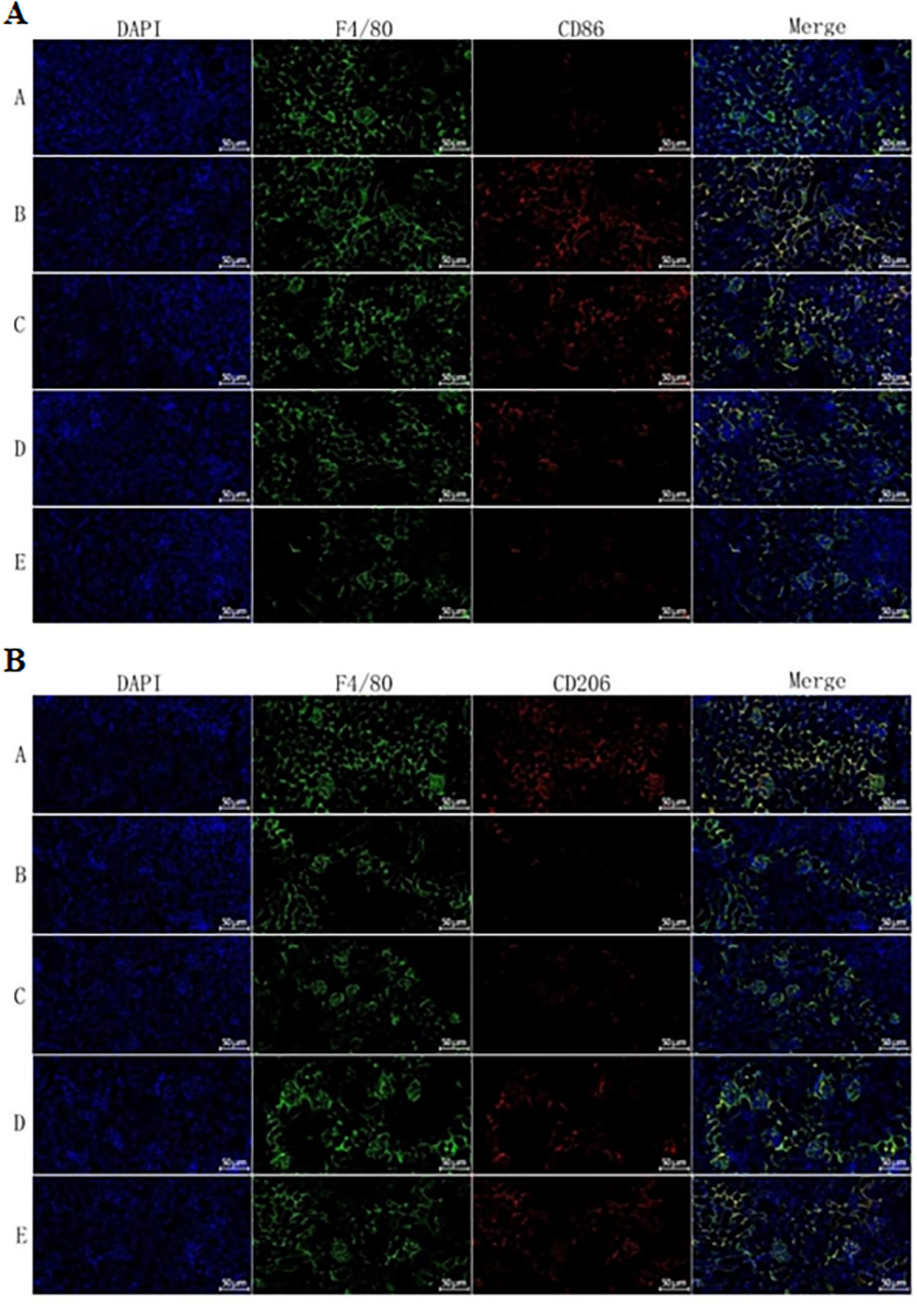
(A) CD86 expression in kidney tissues in the different experimental groups (×200). (B) CD206 expression in kidney tissues in the different experimental groups (×200). Group A, control group; Group B, untreated group; Group C, PRED group; Group D, QJZG group; Group E, 2-DG group. 2-DG, 2-Deoxy-D-glucose group; PRED, prednisone acetate group; QJZG, Qihuang Jianpi Zishen Granules group.

Immunofluorescence analysis was conducted to examine F4/80^+^CD86 and F4/80^+^CD206 expression levels in kidney tissues. In this case, nuclei were stained blue, F4/80+macrophages were labelled green, while CD86+ and CD206+ expressions were marked in red. Quantitative analysis showed that F4/80+CD86 expression was significantly higher while F4/80+CD206 expression was significantly lower in the Untreated group compared with the Control one. Treatment with PRED, QJZG or 2-DG effectively reversed these alterations, leading to a decrease in M1 macrophage markers (F4/80+CD86) and an increase in macrophage M2 markers (F4/80+CD206) relative to the Untreated group (figure 9).

## Discussion

This study demonstrated that mice in the Untreated group exhibited significant renal pathological changes, including glomerulosclerosis, crescent formation, glomerular cell proliferation and extensive inflammatory cell infiltration in the renal interstitium and perivascular areas. Altogether, these pathological features contributed to renal tissue swelling and functional impairment. Additionally, some lupus mice showed signs of renal tubular dysfunction, manifested as renal tubular acidosis and magnesium wasting, thereby resulting in reduced glomerular filtration function. Overall, these findings suggest that MRL/lpr mice can experience both functional and structural alterations in the kidneys. Arg-1 is commonly associated with anti-inflammatory responses, and in this study, its elevated expression in the kidneys of lupus mice may reflect a reduction in inflammation or improved immune regulation.^14^ In contrast, iNOS is closely linked to proinflammatory reactions. Indeed, increased iNOS expression in the kidneys of lupus mice has been associated with inflammatory cell infiltration, elevated pro-inflammatory cytokine levels and oxidative stress.^15^ In this study, the Untreated group exhibited Arg-1 downregulation and iNOS upregulation, indicating that renal pathological changes in MRL/lpr mice could be strongly associated with inflammatory responses and immune dysregulation. Following treatment with QJZG, the renal pathology of MRL/lpr mice was notably improved. In particular, the improvements included partial restoration of glomerular structure and renal tubule alignment, reduced interstitial oedema, decreased inflammatory cell infiltration and significant attenuation of glomerular fibrosis. Furthermore, the upregulation of Arg-1 and downregulation of iNOS following QJZG treatment suggests that these granules may exert their renoprotective effects by suppressing inflammation and promoting renal tissue repair. Additionally, in lupus mouse models, increased ACR and TPCR levels often appeared earlier than other renal function markers, thus making them sensitive indicators for the early detection of renal injury and therapeutic efficacy. Proteinuria is also a common manifestation in lupus mice, and elevated 24-hour PRO levels directly reflect damage to the glomerular filtration membrane. In this study, QJZG treatment significantly improved renal function markers in MRL/lpr mice, suggesting that the granules can alleviate renal injury and may contribute to the functional recovery in LN.

In lupus mouse models, significant alterations in the levels of cytokines and chemokines have been closely associated with disease progression and kidney damage. In this study, it was found that serum TNF-α and IL-1β levels were markedly elevated in MRL/lpr mice, hence highlighting their critical role in SLE-related inflammation as well as their strong correlation with kidney inflammation and tissue injury. Increased IL-12 levels, along with the exacerbation of disease symptoms by exogenous IL-12, also highlight its proinflammatory function in LN. Similarly, elevated IL-23 and IL-27 levels have been linked to inflammation and immune cell activation, with their expression further upregulated under stress conditions.^16 17^ However, TGF-β can exhibit dual proinflammatory and anti-inflammatory properties, thereby acting as a biological switch by modulating the effects of other cytokines or growth factors.^18^ In SLE, TGF-β often enhances local immune responses while demonstrating systemic immunosuppressive effects. This study indicated that TGF-β can shift active inflammatory zones towards areas of resolution and repair, thus mitigating renal inflammation. Furthermore, the elevated levels of IL-4 and IL-10 in treated MRL/lpr mice, compared with the Untreated group, underscored their crucial anti-inflammatory roles in immune regulation. Indeed, IL-4 and IL-10 contribute to immune homeostasis and tissue recovery by suppressing the secretion of proinflammatory cytokines and regulating B cell activity. CCL-18 plays a vital role in immune cell regulation, especially by enhancing regulatory T cell activation, which influences inflammatory responses.^19^ This study revealed that, in MRL/lpr mice, the levels of proinflammatory cytokines TNF-α, IL-1β, IL-12, IL-23 and IL-27, predominantly secreted by M1 macrophages, were significantly increased and strongly associated with inflammation and kidney injury in LN. Conversely, the expression levels of anti-inflammatory cytokines, such as TGF-β, IL-4, IL-10 and CCL-18, which are mainly secreted by M2 macrophages, were markedly reduced, thus suggesting impaired immune regulation. Overall, these alterations not only highlight the pathological mechanism of the disease but also present potential targets for novel therapeutic approaches. Moreover, treatment with QJZG effectively modulated cytokine levels, inhibiting M1 macrophage polarisation while promoting the activation of the M2 phenotype, ultimately ameliorating renal pathology in MRL/lpr mice.

The AMPK/ULK1 signalling pathway plays a critical role in regulating cellular energy metabolism.^20 21^ Indeed, AMPK functions as an intracellular ‘energy sensor’, monitoring fluctuations in ATP levels and modulating metabolic processes by phosphorylating downstream targets. ULK1 is also directly involved in metabolic regulation, and on activation, it phosphorylates key glycolytic enzymes, including HK, phosphofructokinase 1 and enolase 1 as well as fructose-1,6-bisphosphatase in gluconeogenesis.^22^ Under energy stress conditions, AMPK activation triggers ULK1 phosphorylation, which then releases ULK1 from mTOR-mediated suppression. HIF-1α, a critical regulator under hypoxic conditions, modulates the expression of multiple metabolic genes, thereby driving metabolic reprogramming from oxidative phosphorylation to glycolysis.^23^ mTOR activation enhances the transcription and translation of HIF-1α, which subsequently regulates the expression of glycolytic enzymes to ensure cellular energy supply. By suppressing mTOR activity, the AMPK/ULK1 pathway indirectly influences HIF-1α stability, thus modulating metabolic reprogramming.^24^ In summary, the AMPK/ULK1 pathway is essential for maintaining cellular energy balance and redox homeostasis by regulating metabolic reprogramming and interacting with the mTOR/HIF-1α pathway.

CD86 acts as a costimulatory molecule, promoting T cell activation and proliferation through its interaction with CD28 on T cells. iNOS, on the other hand, catalyses the production of NO, a potent proinflammatory mediator that induces oxidative stress and cellular damage. In this study, the elevated expression of CD86 and iNOS in lupus mice could be strongly associated with kidney inflammation and tissue injury. In contrast, M2-type macrophages, especially the M2a subtype, can exhibit high expression of CD206 and Arg-1. In this case, CD206 facilitates the clearance of apoptotic cells and metabolic debris, while Arg-1 metabolises arginine into polyamines and urea, thus contributing to cell proliferation and tissue healing.^25^,^27^ HK2 and GLUT1 are key regulators of glycolysis in M1-type macrophages. Studies have shown^28^ that elevated HK2 expression was strongly correlated with a greater release of proinflammatory cytokines, while GLUT1 overexpression enhanced glucose uptake, supplying metabolic substrates for glycolysis in macrophages. By enhancing glycolysis, HK2 and GLUT1 provide metabolic support for the proinflammatory M1-type macrophages, thus exacerbating kidney inflammation. Overall, targeting HK2 or GLUT1 could serve as a promising therapeutic strategy for LN by regulating macrophage metabolism to reduce kidney damage.

The results indicated that in the kidneys of MRL/lpr mice from the Untreated group, AMPK and ULK1 protein expression was downregulated, with the mRNA levels of AMPK, ULK1 and Atg13 also significantly decreased. In contrast, mTOR mRNA expression was upregulated, suggesting an inhibition of the AMPK/ULK1 pathway. Further analyses revealed an imbalance in M1/M2 macrophage polarisation in MRL/lpr mice kidneys. Notably, the expression of M2-type macrophage markers (CD206 and Arg-1) was significantly reduced at both gene and protein levels, whereas M1-type macrophage markers (CD86 and iNOS) were upregulated. However, QJZG treatment effectively improved renal function parameters and kidney tissue pathology, decreased proinflammatory cytokine levels and increased anti-inflammatory cytokine levels. Additionally, QJZG exhibited dual regulatory effects on macrophage polarisation and metabolic reprogramming in MRL/lpr mice. Renal expression analysis also revealed that QJZG treatment activated the AMPK/ULK1/Atg13 axis while concurrently inhibiting the mTOR pathway. Furthermore, QJZG promoted M2 macrophage differentiation (evidenced by increased CD206 and Arg-1 expression) while suppressing M1 macrophage activation (indicated by decreased CD86 and iNOS expression). Finally, QJZG significantly attenuated glycolytic activity in M1 macrophages by downregulating HK2 and GLUT1 expression.

While this study demonstrates the potential beneficial effects of QJZG in a murine model of lupus, several limitations should be acknowledged. first, all experiments were conducted within a single animal model; although QJZG is already used clinically in China, further validation in other experimental models and studies incorporating human-derived materials remains necessary to better anticipate its potential translational value. Additionally, similar to prednisone and 2-DG, the improvement conferred by QJZG was only partial, as parameters in all treatment groups, despite being significantly improved compared with the untreated group, still did not reach the levels of the healthy controls. Furthermore, the current experimental approach did not identify a distinct immunological mechanism uniquely attributable to QJZG, nor one that clearly differentiates it from glucocorticoid or 2-DG-mediated effects. Thus, this work cannot yet clarify the more specific role of QJZG in the treatment of autoimmune diseases. Future studies employing more diverse models and deeper mechanistic investigations will be essential to elucidate the precise mode of action and potential synergistic or unique therapeutic properties of QJZG.

## Conclusions

Together, our results demonstrate that QJZG mitigates renal injury in MRL/lpr mice. This therapeutic effect was accompanied by a shift in macrophage polarisation from the M1 to the M2 phenotype and a reduction in proinflammatory cytokines. Furthermore, we observed that QJZG induced activation of the AMPK/ULK1 pathway, which is a known regulator of macrophage polarisation, suggesting a potential mechanistic involvement of this pathway in the observed immunomodulatory and renoprotective effects.

## Supplementary material

10.1136/lupus-2025-001639 online supplemental file 1

## Data Availability

All data relevant to the study are included in the article or uploaded as supplementary information.
